# Equivalence Between Optical Flow, the Unrest Index, and Walking Distance to Estimate the Welfare of Broiler Chickens

**DOI:** 10.3390/ani15091311

**Published:** 2025-05-01

**Authors:** Danilo Florentino Pereira, Irenilza de Alencar Nääs, Saman Abdanan Mehdizadeh

**Affiliations:** 1 Department of Management, Development and Technology, School of Science and Engineering, Sao Paulo State University, Tupã 17602-496, SP, Brazil; 2 Graduate Program in Production Engineering, Paulista University—UNIP, São Paulo 04026-002, SP, Brazil; irenilza.naas@docente.unip.br; 3 Department of Mechanics of Biosystems Engineering, Faculty of Agricultural Engineering and Rural Development, Agricultural Sciences and Natural Resources University of Khuzestan, Ahvaz 63417-73637, Iran; saman.abdanan@gmail.com

**Keywords:** machine vision, movement analysis, precision livestock farming, unrest index, YOLO

## Abstract

Modern poultry production demands scalable and non-invasive methods to monitor animal welfare, particularly as broiler strains are increasingly bred for rapid growth, often at the expense of mobility and health. This study evaluates two advanced computer vision techniques—Optical Flow and the Unrest Index—to assess movement patterns in broiler chickens. Three commercial broiler strains (Hybro^®^, Cobb^®^, and Ross^®^) were housed in controlled environments and continuously monitored using ceiling-mounted video systems. Chicken movements were detected and tracked using a YOLO model, with centroid data informing both the Unrest Index and distance walked metrics. Optical Flow velocity metrics (mean, variance, skewness, and kurtosis) were extracted using the Farnebäck algorithm. Pearson correlation analyses revealed strong associations between Optical Flow variables and traditional movement indicators, with average velocity showing the strongest correlation to walked distance and the Unrest Index. Among the evaluated strains, Cobb^®^ demonstrated the strongest correlation between Optical Flow variance and the Unrest Index, indicating a distinct movement profile. The equipment’s movement and the camera’s slight instability had a minimal effect on the Optical Flow measurement. Still, its strong correlation with the Unrest Index and walking distance accredits it as an effective method for high-resolution behavioral monitoring. This study supports the integration of Optical Flow and Unrest Index technologies into precision livestock systems, offering a foundation for predictive welfare management at scale.

## Simple Summary

Rapid growth in modern broiler production often compromises mobility, highlighting the need for effective, non-invasive welfare assessment tools. This study compares two vision-based methods (Optical Flow and the Unrest Index) for detecting behavioral indicators of lameness and inactivity. Three commercial broiler strains (Hybro^®^, Cobb^®^, and Ross^®^) were continuously monitored using YOLOv8m for bird detection. Movement metrics derived from Optical Flow (mean, variance, skewness, and kurtosis) were analyzed alongside the Unrest Index and walking distance. Results revealed strong correlations between these indicators and strain-specific behavioral patterns. These findings demonstrate the potential of Optical Flow and the Unrest Index as automated, scalable solutions for continuous welfare monitoring, offering practical applications within Precision Livestock Farming systems.

## 1. Introduction

Animal welfare is a growing global concern, as are public ethics, food security, and sustainable production. In broiler chicken systems, welfare impairments often manifest as mobility issues, such as gait instability, leg disorders, and inactivity, compromising health and productivity [1,2]. Assessing welfare at scale in these high-density environments remains challenging, especially when traditional individual-based evaluations are neither feasible nor timely [3,4].

Animal welfare is about how animals feel, ensuring that they experience minimal suffering and high positive emotions. To properly assess their well-being, it is essential to understand these internal states [5]. However, we cannot directly measure feelings; therefore, we rely on indirect methods to infer what the animal is experiencing. Preference and motivational tests provide insights into animals’ choices and the strength of their preferences [6]. Physiological indicators of compromised health and stress responses offer valuable corroborating evidence of impaired welfare [7,8]. Vučinić and Lazić [9] explored the definition of animal welfare and various assessment methods, emphasizing the role of feeding, housing, health, and appropriate behavior in influencing animal welfare.

Animal behavior is a crucial tool for assessing animal welfare, both in research and on farms. It helps us understand animal health and identify needs, especially when combined with new technologies and preference tests [1,5]. Behavioral diversity is increasingly used as an indicator of welfare. Studies suggest that animals exhibiting a wide range of natural behaviors experience better welfare, while those restricted to fewer behaviors might be under stress or discomfort [10,11]. However, this approach requires careful interpretation, as aggressive behaviors, restlessness, and dominance do not contribute to the flock’s welfare and must be considered in isolation. The Five Domains Model approach, involving nutrition, environment, health, behavior, and sentience, underscores the significance of the ability to assess positive welfare states by incorporating an animal’s capacity for choice, control, challenge, agency, and behavioral interactions. This attempt enables a more holistic evaluation of animal well-being, moving beyond a singular focus on physiological parameters [10,11,12,13].

Natural behavior detection addresses the challenges of detecting and tracking individual chickens and classifying natural behaviors. New deep learning-based models, such as YOLO, have brought about significant advances in the detection and tracking of broiler chickens [14,15,16]. However, tracking remains challenging due to the high-density conditions typically found in commercial farming systems, where birds are often crowded together, and equipment causes occlusions in the images, making it challenging to maintain the identification of chickens over time [17,18]. Group behaviors are essential and do not require animal identification. Flock behavior can be assessed through multiple methodologies, including acoustic monitoring [19,20], thermal imaging [21], and the use of video cameras [22,23].

Walking ability is increasingly recognized as a critical indicator of animal welfare, particularly in broiler chickens. Impaired walking ability is linked to numerous welfare issues, including pain, restricted access to food and water, and an inability to express natural behaviors [24,25]. Automated gait analysis has been shown to correlate with other welfare measures, such as leg health, and it offers the potential for more efficient monitoring in large-scale animal production systems [3,26]. These objective assessments are crucial in modern farming, where welfare monitoring must be efficient [1]. However, modern broiler production systems house thousands of birds, making traditional individual-based monitoring inefficient and impractical [27], as these flocks’ activity levels can provide essential information on animal welfare outcomes.

Among the computer vision methods applied to standard video camera images, two widely tested approaches in experimental settings stand out: Optical Flow [28] and the Unrest Index [29]. The two methods use different strategies and resources to obtain metrics of body displacement in videos. While Optical Flow uses statistical methods to monitor the variation in brightness between frames and thus obtain the average, variance, skewness, and kurtosis metrics to summarize the displacement in time (speed), the Unrest Index uses the Hausdorff distance, which is a mathematical method, to calculate the distance that represents the displacement of the entire group of animals between two frames. Both methods depend on the frame rate of the camera used to capture the images since the higher the image capture rate, the smaller the variation in the position of the bodies in the images, and therefore, the smaller the measurements obtained. In this case, both techniques depend on standardizing the frame rate to be processed by the methods.

Optical Flow, which analyzes apparent motion between video frames, is increasingly applied in precision livestock farming to monitor broiler movement. While this technique has seen broad application in fields such as robotics [30,31,32], medical imaging [33,34], and fluid dynamics [35,36], its relevance in poultry lies in its ability to capture flock-level motion patterns without individual identification, enabling automated welfare assessments [23,27]. Previous studies have shown that Optical Flow metrics such as the mean, variance, skewness, and kurtosis of movement distributions correlate with crucial animal welfare indicators like mortality, the gait score, and the prevalence of leg disorders such as hock burns and pododermatitis [22,37]. These metrics allow producers to infer flock-wide health trends from video data, offering a non-invasive and automated approach to welfare assessments [38]. However, establishing a direct and clear link between flock-level Optical Flow patterns and the welfare of individual birds remains a complex endeavor [39,40].

Del Valle et al. [29] proposed the Unrest Index using video images. Based on the Hausdorff distance measure, the index was evaluated using video recordings of chickens from various experimental settings. The index effectively detected signs of different movement patterns in poultry under different thermal conditions, indicating its sensitivity to changes in environmental stressors. Pereira et al. [41] used the Unrest Index to evaluate the gait of broiler chickens, while Fernandes et al. [42] applied it to estimate the welfare of laying hens raised under different lighting sources and thermal conditions.

In deep learning, YOLO models have been widely used for animal detection in complex environments. In poultry farming, several studies have successfully applied it to chicken detection [43,44]. The method has also been used to track individual chickens [14,15,16,45]. Despite the high computational cost of training a YOLO model for chicken detection and tracking, this appears to be the most accurate method for assessing bird movement in complex environments. All three methods for evaluating the movement of chicken flocks described have been widely tested, but there has not yet been a comparison between these methods. To what extent are the measurements from these methods equivalent? Is it possible to replace one method with another, depending on the availability of computational resources or the quality of the sensor and the images collected?

These questions are essential because the conditions of poultry farms may not be conducive to replicating a given method on farms. Lighting conditions and image resolution resulting from camera quality may not favor using a given technique. The unavailability of better computational resources or the difficulty of training YOLO-based detection models may restrict their use. Therefore, knowing the equivalence of the metrics provided by the methods is essential for this field of knowledge and poultry farming, which can benefit from these tools for flock monitoring.

This study hypothesizes that the metrics derived from the Unrest Index, YOLO-based tracking, and Optical Flow are equivalent, providing robust indicators of flock movement for the early detection of welfare issues. Confirming this hypothesis will lay the foundation for integrating vision-based tools into commercial broiler management systems. This study aimed to verify the equivalence of metrics from three computer vision methods: (1) the Unrest Index, which calculates restlessness from the displacement of the body’s geometric centers (centroids) between frames; (2) the average walking distance measured using YOLO-based detection and tracking; and (3) Optical Flow, which measures the speed of frame-to-frame displacement of pixel clusters. The methods were tested on images from an experiment monitoring three broiler breeds. Confirming the equivalence of these methods will ensure robust movement indicators for early welfare problem detection and support the integration of vision-based tools in future broiler management.

## 2. Materials and Methods

### 2.1. Experimental Setup

Broiler breeders from three commercial strains (Hybro^®^, Cobb^®^, and Ross^®^), aged 29, 26, and 32 weeks, were housed simultaneously and separately in three pens within a climate chamber. Each pen contained twelve birds (ten females and two males) and was equipped with a designated nesting area, a suspended feeder, and a drinker. Feed allocation was controlled and calculated based on the breeders’ productive phase, mirroring commercial broiler breeder housing systems. The experiment lasted nine days, and the temperature was controlled, starting at 24 °C for the first three days, increasing to 28 °C for the following days, and ending at 32 °C for the last three days. This study was conducted following standard husbandry practices and in compliance with Directive 2010/63/EU on protecting animals used for scientific purposes. All animal handling and care procedures complied with the established welfare guidelines for poultry in commercial production systems.

The continuous monitoring of the birds’ movements was facilitated by RGB video cameras (Mythos^®^, Hikvision, Hangzhou, China) with a resolution of 420 lines, which were equipped with a 3.6 mm lens and a capture rate of 16 fps mounted on the ceiling of the pens and directed downward to capture the entire area. To ensure accurate image processing, irrelevant background regions, such as walls, pipes, and windows, were excluded. These areas, characterized by high brightness, could interfere with binarization. The impact of irrelevant regions on the results was minimized by selecting and clipping only the places of interest in all frames of the analyzed videos. The dataset used in this study consisted of 15 videos of approximately 15 min, always filmed in the afternoon, totaling approximately 6 h of footage. Sample of the video and dataset is available in the Supplementary Material.

### 2.2. Walking Distance Assessment

The centroid of the bird’s bounding box was determined by detecting the chicken using YOLO. The Euclidean distance traveled between two sequential frames from these coordinates was summed until the chicken’s identification was lost.

(1) $$d_{ID}=\sum_{i=f2}^{fn}\sqrt[{2}]{{\left(x_{i}-x_{i-1}\right)}^{2}+{\left(y_{i}-y_{i-1}\right)}^{2}}$$

where $d_{ID}$ is the distance walked by an identified chicken (ID), $\left(x_{i},y_{i}\right)$ are the chicken centroid coordinates in the actual frame, $\left(x_{\left(i-1\right)},{y\;}_{\left(i-1\right)}\right)$ are the coordinates of the same chicken in the previous frame, f2 is the second frame where the chicken was identified, and fn is the last frame where the bird was identified.

The potential loss of bird identification during the tracking process did not interfere with the analysis. For each video processed, the total distances walked by all identified birds were summed and compared with those obtained using the other methods.

### 2.3. The Unrest Index Method

The process begins by determining the distances between all the centers of the birds’ bounding boxes in consecutive frames, specifically the version used to calculate the Unrest Index (measured in pixels). From these measurements, the Restlessness Index is calculated using the Hausdorff distance as a basis, as described by [29] in Equation (2). The calculated values of the Unrest Index were stored in an .xls file.

(2) $${Unrest\;Index}_{\left(i,i-1\right)}=max\{dH\left(F_{\left(i\right)},F_{\left(i-1\right)}\right),dH\left(F_{\left(i-1\right)},F_{\left(i\right)}\right)\}$$

where the ${Unrest\;Index}_{\left(i,i-1\right)}$ is the movement of the birds between two frames, i is the position of the frame in the video, $F_{\left(i\right)}$ is the current frame, $F_{\left(i-1\right)}$ is the previous frame, and dH is the Hausdorff distance between a group of birds from one frame to another. YOLO detection was used to find the centroids of birds in the scenes. However, the Unrest Index can use any segmentation technique to detect birds in the frames since what feeds this model are only the coordinates of the centroids.

### 2.4. YOLO Model for Detecting the Chickens

**Figure 1:**
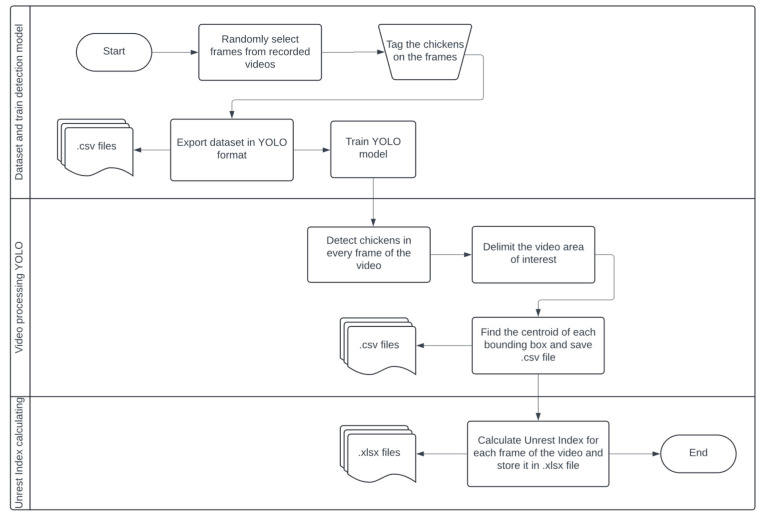
Flowchart of the steps in detecting chickens with YOLOv8m and calculating the Unrest Index.

Figure 1 presents the steps for detecting chickens using the YOLOv8m version and calculating the Unrest Index. YOLO popularized regression algorithms for object detection [38]. While detecting the chickens, the centroids were recorded for later calculation of the Unrest Index [29].

A meticulously curated dataset was constructed to train a deep-learning model for detecting chickens in the video footage. A total of 57 frames were randomly selected from over six hours of footage. Using the Label Studio tool [39], all chickens within these frames were annotated with bounding boxes that precisely defined their locations. The bounding box coordinates were subsequently exported to the standardized Yolo format for object detection tasks. The labeled dataset was rigorously divided into three subsets to facilitate a robust, generalizable model. The primary training set, comprising 70%, was the foundation for model learning. A validation set (15%) was crucial in optimizing training by providing a platform for performance evaluations and hyperparameter adjustments.

Finally, the unseen test set (15%) was the ultimate benchmark for evaluating the model’s ability to detect chickens in real-world scenarios. The Yolov8m architecture was used to detect chickens in videos. The model was trained with 300 epochs on an Intel i7 computer (10,700 K) (Intel Corporation, Santa Clara, CA, USA) with 64 GB of RAM, an 8 GB NVIDIA Geforce RTX2070 Super GPU (NVIDIA Corporation, Santa Clara, CA, USA), and the Windows 10 operating system. The effectiveness of the trained model in detecting chickens within the video footage was assessed using a variety of performance metrics:

(3) $$P=\frac{TP}{TP+FP}\cdot 100\%$$

(4) $$R=\frac{TP}{TP+FN}\cdot 100\%$$

(5) $$F1=\frac{2\cdot Precision\cdot Recall\;\;}{Precision+Recall\;}\cdot 100\%$$

(6) $$AP=\int_{0}^{1}P\left(R\right)dR\cdot 100\%$$

(7) $$mAP=\frac{1}{n}\sum_{k=1}^{n}{AP}_{k}\cdot 100\%$$

where P = precision, R = recall, TP = true positives, TN = true negatives, FP = false positives, FN = false negatives, AP = accuracy of a particular class, mAP = average accuracy of all classes, and n: number of detected classes, which in our case equals 1 (chicken).

This study did not aim to create a generalized chicken detection model for application in other videos. This model should be able to detect the chickens accurately in the videos evaluated to compare the methods. Therefore, the overfitting characteristics of this model do not represent a problem for this study.

The pixel values within the video frames were accumulated during the processing phase to accurately measure the distance the birds traversed. Concurrently, the coordinates of the centroids of the birds’ bounding boxes were recorded and stored in .csv files for later calculation of the Unrest Index.

### 2.5. Optical Flow Method

Optical Flow is used to detect motion patterns. Such an analysis involves detecting the intensity differences in each area of an image frame both through time and space [46]. Optical Flow can be detected at the pixel level; however, each image frame in the video is divided into 8-by-8-pixel blocks for improved processing efficiency. The squared Euclidean distance between the two sub-windows was defined to identify a displacement of the pattern in the ith image [38]. The spatial mean, variance, skewness, and kurtosis of the estimated flow displacement over the image were calculated frame by frame [23]. Four Optical Flow measures (mean, variance, skew, and kurtosis) were collected from each sequence of image frames, representing a real-time block.

The Farnebäck method was chosen for the Optical Flow assessment. The Farnebäck algorithm provides dense Optical Flow, which calculates motion at each pixel rather than only specific feature points [47].

Each corresponding pair of sub-windows from the first–second image was compared. Let $I_{1}^{i,j}$ denote the sub-window at position (*i*,*j*) in the first image, and $I_{2}^{i,j}$ denote the corresponding sub-window in the second image. The objective was to determine the presence of any discernible pattern within $I_{1}^{i,j}$. To achieve this, the squared Euclidean distance between the two sub-windows was calculated (Equation (8)):

(8) $$R_{e}(s,t)=\sum_{m=0}^{M-1}\sum_{n=0}^{N-1}{\left[I_{1}^{i,j}\left(m,n\right)-I_{2}^{i,j}\left(m-s,n-t\right)\right]}^{2}$$

For every possible overlap between the sub-windows, the sum of the squared difference was calculated to identify the position where the sub-windows exhibited the least dissimilarity. Expanding the square term in Equation (7) yields Equation (9):

(9) $$R_{e}\left(s,t\right)=\sum_{m=0}^{M-1}\sum_{n=0}^{N-1}{\left[I_{1}^{i,j}\left(m,n\right)-I_{2}^{i,j}\left(m-s,n-t\right)\right]}^{2}\;=\sum_{m=0}^{M-1}\sum_{n=0}^{N-1}I_{1}^{i,j}{\left(m,n\right)}^{2}-{2I}_{1}^{i,j}\left(s,t\right)\cdot I_{2}^{i,j}\left(m-s,n-s\right)+I_{2}^{i,j}{\left(m-s,n-t\right)}^{2}$$

It is evident that the first term, $I_{1}^{i,j}{\left(m,n\right)}^{2}$, remains constant as it is independent of the displacement variables *s* and *t*. Similarly, the last term, $I_{2}^{i,j}{\left(m-s,n-t\right)}^{2}$, depends solely on the second image and is, therefore, constant with respect to s and t. Consequently, the only term that varies with s and t is the middle term, which involves the product of corresponding pixel intensities from both images. This term, excluding the factor of −2, is commonly referred to as the cross-correlation (or circular cross-correlation) and is defined as

(10) $$C(s,t)=\sum_{m=0}^{M-1}\sum_{n=0}^{N-1}I_{1}^{i,j}\left(m,n\right)\cdot I_{2}^{i,j}\left(m-s,n-t\right)$$

### 2.6. Delimited Area of Interest for the Videos

**Figure 2:**
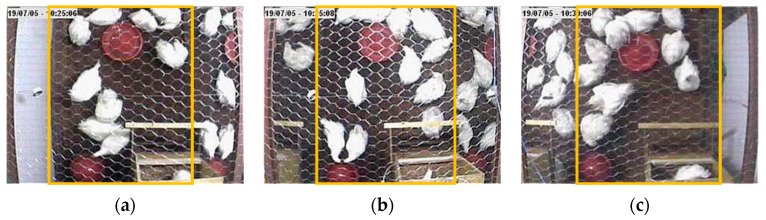
Delimited areas of the pens housing the Hybro (**a**), Cobb (**b**), and Ross (**c**) commercial strains, which were used for video processing and data extraction across different methods for estimating chicken movement ability.

The trained Yolo model was applied to all videos. The strains were raised in pens side by side so that birds from another strain appeared in the video of one strain. So that the distance, the Unrest Index, and Optical flow data were exclusive to the strain of interest, we delimited the area for data processing and extraction. Figure 2 shows the upper view of the pens with the three commercial strains researched.

### 2.7. Statistical Analysis

Totals and averages calculated from the videos per experimental day were compared. A linear regression analysis was performed on the data concerning distances walked and the Unrest Index to demonstrate the equivalence of these two methods for estimating chicken movement within the housing environment. The results obtained from the Optical Flow method were compared with the distance walked and Unrest Index measurements using Pearson correlation analysis.

## 3. Results

### 3.1. YOLO Chicken Detection Model

**Figure 3:**
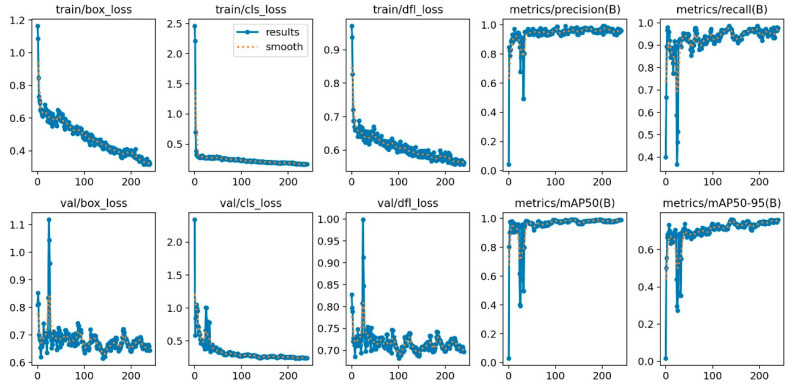
Graph of the evolution of performance metrics during the training and validation of the object detection model over 300 epochs.

Figure 3 presents the performance metrics, including Training Bounding Box Loss (train/box_loss), Training Classification Loss (train/cls_loss), Training Objectness Loss (train/obj_loss), Precision for Class B (metrics/precision (B)), Recall for Class B (metrics/recall (B)), Validation Bounding Box Loss (val/box_loss), Validation Classification Loss (val/cls_loss), Validation Objectness Loss (val/obj_loss), Mean Average Precision at IoU = 0.50 for Class B (metrics/mAP50 (B)), and Mean Average Precision at IoU = 0.50:0.95 for Class B (metrics/mAP50-95 (B)).

### 3.2. Comparison of Walking Distances and Unrest Index

**Figure 4:**
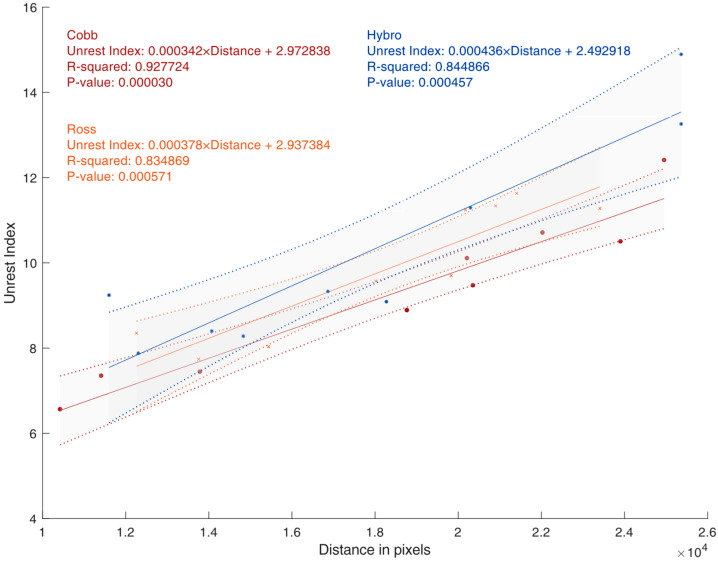
Linear regressions and band of standard error of the values obtained for the total distance walked by all chickens on each day of the experiment (morning + afternoon) with the calculated Unrest Index values.

The standard unit of measurement (pixels) for distance walked and the Unrest Index permits a direct comparison. Figure 4 shows the linear regression results for the three evaluated strains. The results show that the distance walked measure and the Unrest Index have a strong correlation and can be used to estimate the movement of birds in a flock. The analysis of the R^2^ of the equations allows us to state that there is a linear correlation between the distances walked by chickens and the Unrest Index.

### 3.3. Comparison of Walking Distance, Unrest Index, and Optical Flow Methods

**Figure 5:**
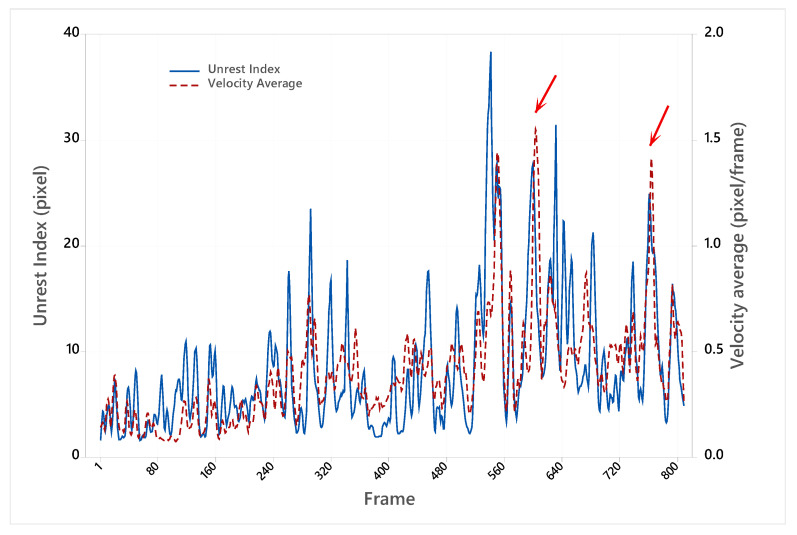
Temporal variation in the Unrest Index and average Optical Flow speed in a Cobb^®^ video recorded during the afternoon session. Red arrows indicate peaks of flock activity observed in both approaches.

Figure 5 shows the frame-by-frame variation in the Unrest Index and the average Optical Flow speed obtained for an example video of the Cobb^®^ strain in the afternoon. It is possible to verify that chicken agitation peaks at very close times in both methods.

The intensities and precise moment of changes in the Unrest Index and the speed measured by the Optical Flow method are not precisely the same due to the specificities of the techniques used to obtain the values. Both methods efficiently determine chicken movements at the time intervals, such as the 6 min videos used in this work (Table 1).

Pearson correlation analysis indicates strong associations between Optical Flow variables, the Unrest Index, and distances traveled measured by Yolo. Notably, the average speed exhibited a strong positive association with the distance traveled and the Unrest Index. Specifically, the Optical Flow average emerged as the variable most strongly correlated with the distance traveled and the Unrest Index. In Cobb^®^, the variance of Optical Flow had a higher predictive value than the average speed. These findings suggest that Optical Flow analysis can effectively capture critical aspects of broiler behavior, including activity levels and movement patterns.

### 3.4. Advantages Between Walking Distance, Unrest Index, and Optical Flow Methods

The Optical Flow analysis was the simplest to apply. While the calculation of the distances walked by the YOLO method required a labeling effort to generate a robust dataset and the computational effort for training, the calculation of the Unrest Index was performed in two stages, the first being the identification of the centroids (in this work, the already trained YOLO model was used) and then the calculation of the metric of interest; the Optical Flow method eliminates the need for labeled training data or individual detection steps. However, this method may have inferred the movement of the chickens with more significant inaccuracy.

**Figure 6:**
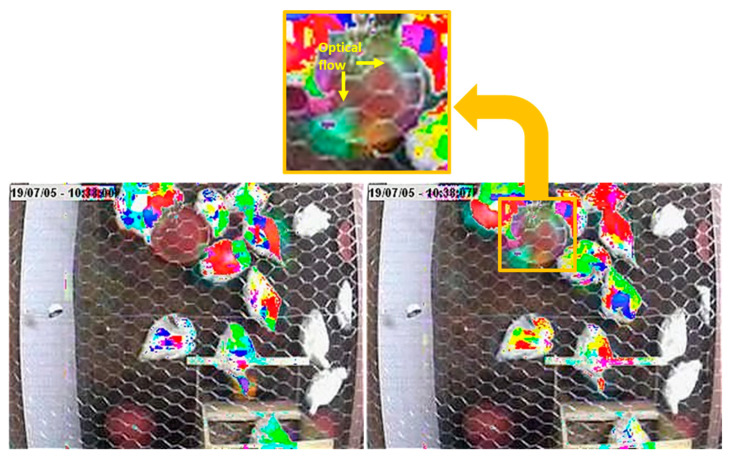
The movement of the feeder in the analysis of two sequentially captured frames and the highlighted Optical Flow method.

Upon analyzing the processed videos, we found that the camera was not perfectly stabilized. This slight camera movement is primarily evidenced in the Optical Flow results, where several fixed parts of the scene exhibit sudden changes in brightness. In addition to the lack of camera stability, the pendulous movement of the feeder and waterer and the time count in the left corner of the video contributed to this issue. These variations in brightness were detected by the Optical Flow model, thereby affecting its variable values. The movement of the feeder in two sequential frames, as captured by the Optical Flow analysis, is highlighted in Figure 6. Although the Optical Flow results captured unwanted scene elements (e.g., feeder motion), the method demonstrated a robust correlation with movement-based welfare indicators.

The lack of camera stability is also evident in the nest’s perch, which, although fixed, exhibits variations in brightness as captured by the Optical Flow method. In this approach, several environmental elements contributed to the average Optical Flow speed between frames. In contrast, these environmental elements did not affect the YOLO-based approach, which focused on detecting and tracking individual chickens.

Nevertheless, despite these limitations, a strong correlation was observed between the detachment speeds obtained from Optical Flow, the measured distances walked, and the chickens’ Unrest Index. Such a result suggests that Optical Flow analysis can be a valuable tool for evaluating the walking ability of the chicken flock, even in the presence of environmental noise. Table 2 summarizes the advantages and disadvantages of each method assessed.

## 4. Discussion

The Optical Flow, Unrest Index, and walking distance measurements showed strong correlations, demonstrating equivalence for using these methods in monitoring the movement of chicken flocks. Walking distance is the most accurate method if the chicken detection and tracking model is precise. With this method, it is possible to detect clustered individuals accurately, and tracking allows us to know the movement of each individual. Although YOLO tracks individual chickens [14,15,16], the equipment occlusion of birds causes identification to be lost. In this study, a summary of the walking distance data obtained in YOLO was made, representing the group, so this measurement was compared and equated with the Optical Flow and Unrest Index metrics.

However, solving the problem of bird occlusion by equipment or even the loss of identification due to model confusion is challenging [15,17]. Mehdizadeh et al. [18] worked on this issue by using image segmentation to minimize these identification losses during the tracking process.

The Unrest Index is a method that does not aim to monitor the movement of each chicken but to extract a displacement measurement that represents the flock frame by frame. In this proposal, tracking the bird is not relevant, and only its detection is important, which makes it very accurate in its proposal when combined with the YOLO method for bird detection. In this method, it is also possible to apply other image-processing techniques to segment the chickens and determine the centroids, making it a more flexible method for use in cases where computational resources are limited.

Optical Flow was the simplest method used. There is no need to propose a previous training or detection step for the birds. The videos were processed according to the original recordings, and the metrics were easily extracted. This method provides four statistical metrics (average, variance, skewness, and kurtosis) that describe the data distribution on the movement speed of the bodies in the video. The metric that most correlated with walking distance and the Unrest Index was the average. Still, the variance, skewness, and kurtosis provide relevant information about the flock’s movement, as the higher these values are, the more individuals with different speeds move in the video.

All methods had an error resulting from frame-by-frame processing. While the Optical Flow metrics are affected by the lack of camera stabilization and the movement of other objects in the scene, walking distance identifies a more significant number of animals in the confined environment due to identification losses and restarts tracking the same animal, resulting in shorter walking distances than those that occurred. The Unrest Index depends on bird detection, and although it suffers less from the effects of identification loss when the chicken is completely hidden behind some equipment, the loss of that centroid between frames and its reappearance in the next frame increases the value of this metric. However, all methods are sufficiently accurate for automatically monitoring chicken movement.

Previous studies have shown correlations between flock Optical Flow and average individual behavior through specific tests like runway and water tests [22]. However, the precise individual behavioral mechanisms that give rise to these flock-level patterns and their direct causal relationship with various welfare states are not fully understood [40].

The primary advantage of using Optical Flow in broiler management lies in its computational simplicity and scalability. By detecting changes in brightness across successive frames, Optical Flow algorithms can capture collective behavior patterns within large groups of birds [48]. This methodology enables real-time monitoring of broiler movements and behaviors, identifying deviations from expected activity levels that may indicate health or welfare issues [23]. Flocks with poor welfare outcomes tend to exhibit reduced movement, increased variance in Optical Flow, and a higher percentage of birds displaying abnormal walking behavior [22]. These findings underscore the potential of Optical Flow as a powerful tool for the early detection of welfare problems, allowing for timely interventions to improve flock health and productivity [27].

As the poultry industry increasingly adopts precision livestock farming technologies, integrating computer vision and big data analytics will further enhance the predictive capabilities of Optical Flow-based systems. Advances in machine learning and deep learning algorithms will allow for more accurate analysis of complex movement patterns, facilitating a shift toward predictive rather than reactive flock management [37,38]. This transition will enable poultry producers to monitor and anticipate welfare issues, contributing to a more sustainable and ethical approach to broiler production [47].

The bird’s unimpeded movement, lacking any signs of lameness, is a crucial indicator of its overall health, reflecting the optimal functioning of a well-maintained physical form. An animal’s voluntary active participation demonstrates motivation and a sense of well-being [1,37]. Consequently, active locomotion can be a reliable indicator of physical well-being and positive emotional welfare.

When comparing the methods, we found the effectiveness of the Unrest Index and Optical Flow methods in detecting movement and correlating it with welfare indicators. Traditional welfare assessments apply the walking distance to infer welfare metrics like gait scores and walking distances, implying that the bird moving, as usual, is in good health [23]. These methods’ computational simplicity, non-invasiveness, and relevance favor modern farming practices associated with precision livestock farming [27].

The ability to continuously monitor movement and activity levels allows for the early identification of leg disorders, pododermatitis, or hock burns. These conditions often correlate with decreased mobility and increased inactivity in broiler chickens [26]. By identifying abnormal movement patterns early, these methods enable timely interventions, such as adjustments to feeding, housing, or temperature, which can prevent the progression of these issues. Leg disorders, a prevalent welfare concern in broilers, are often linked to rapid growth rates and poor walking ability. The present study demonstrates that walking ability, captured through movement metrics like the Unrest Index and Optical Flow, correlates with the birds’ health [22]. The early detection of reduced mobility supports interventions that reduce pain and prevent the progression to severe leg disorders, thus improving health outcomes.

Automated systems can alert farm managers to potential welfare issues in real time, providing opportunities for quick interventions. This proactive approach can prevent the escalation of welfare problems, leading to healthier flocks and improved productivity [47]. Van der Eijk et al. [40] demonstrated high movement detection using Mask R-CNN, which was trained on mixed data, and robust resource tracking via zone classifiers, suggesting a scalable, real-time solution for commercial farms.

Integrating Optical Flow, Unrest Index techniques, and machine learning algorithms can revolutionize precision livestock farming. Optical Flow offers the advantage of real-time analysis without extensive model training. Valuable insights into flock behavior can be extracted by calculating the four variables (mean, variance, skew, and kurtosis) from the recorded videos. The variables could be stored efficiently while the image data can be discarded, reducing storage requirements. In contrast, the Unrest Index requires a prior step for centroid localization, and once obtained, it also discards the original images. Implementing a strategy for automated dataset expansion is crucial to ensure the model’s continued accuracy and adaptability.

A multi-faceted strategy can be employed to harness the strengths of both approaches. Initially, Optical flow algorithms will provide a foundational understanding of overall flock movement patterns. Subsequently, a more granular analysis will be conducted using the Unrest Index and other relevant metrics derived from the YOLO object detection framework. This combined methodology will comprehensively assess flock behavior, capturing subtle and overt movement dynamics.

These methods and data analytics could shift welfare management from reactive to predictive, allowing for more proactive approaches to animal health [38,47]. Technologies like Optical Flow contribute significantly to animal welfare assessments by providing a non-invasive, continuous evaluation of their movement behavior. This approach aligns with welfare standards such as the Five Domains Model, which expands traditional welfare assessments beyond the physical to include emotional and mental states [12]. By enabling real-time monitoring of movement and behavior, Optical Flow can detect signs of discomfort, stress, or pain without requiring intrusive procedures like manual handling, which can cause additional stress to the animals.

Thermal stress has been extensively documented to have detrimental effects on poultry welfare. Elevated temperatures can lead to heat stress, which disrupts normal physiological functions, impairs immune responses, and leads to oxidative stress [8]. Heat-stressed birds often show lethargy, reduced feed intake, and diminished movement, negatively impacting their welfare. Prolonged exposure to heat stress can result in higher mortality rates, lower productivity, and an increased incidence of welfare issues like leg disorders, as inactivity exacerbates conditions like pododermatitis and hock burns [4,26].

In the present study, all three methods have the potential to provide real-time data on movement patterns, enabling the identification of reductions in activity caused by high environmental temperatures. These technologies allow for the early detection of thermal stress, which is linked to welfare outcomes like decreased walking ability and leg health issues. Effective environmental management strategies, particularly those focused on temperature control, play a crucial role in mitigating the negative impacts of thermal stress. Proper ventilation, temperature regulation, and housing design can help maintain conditions within the birds’ thermoneutral zone, thus reducing the likelihood of thermal stress and associated behavioral changes. Ventilation systems that provide consistent airflow can help dissipate excess heat, while automated temperature control systems can adjust heating and cooling mechanisms based on real-time environmental data.

Previous research shows that environmental adjustments, such as improved ventilation or cooling systems (e.g., evaporative cooling), can alleviate thermal stress and promote more natural behavior in broilers, including increased movement and feed intake [21]. Additionally, providing shaded areas, adjusting stocking densities, and optimizing lighting conditions further support broiler welfare by minimizing environmental stressors that lead to unrest or abnormal behaviors [20]. The strains (Hybro^®^, Cobb^®^, and Ross^®^) exhibited different behaviors, and these differences in walking patterns could inform selective breeding strategies to enhance welfare outcomes [3].

The large volumes of data generated by machine vision systems can be integrated with big data analytics and machine learning algorithms to improve decision-making in poultry management [38]. Machine learning models can be trained to recognize patterns in flock movement data that correlate with specific welfare outcomes, enabling more accurate predictions and interventions. For instance, predictive models could be developed to flag potential health issues based on movement pattern deviations, providing farm managers with actionable insights [49,50]. This represents a significant step toward data-driven precision livestock farming, where welfare management is continuously optimized using advanced computational tools [47].

All three methods leverage inexpensive camera equipment and do not require sophisticated sensors or invasive tracking systems [29,43]. These methods make it affordable for farmers to implement automated welfare monitoring systems. In addition, as technology advances, the cost of computational power required to process images is expected to decrease, making it even more accessible to a broader range of poultry producers [38]. The combination of low-cost hardware and robust software analysis renders all three evaluated methods highly cost-effective solutions for enhancing welfare monitoring in poultry production.

The Unrest Index is sensitive to temperature changes; however, further research could refine its application in varying environmental conditions. Future research should refine the Unrest Index and Optical flow algorithms, particularly in minimizing the impact of the artifacts (e.g., camera stability and feeder movement) and improving sensitivity to welfare changes.

The application of Optical Flow analysis holds significant promise for the automated and continuous assessment of broiler welfare in commercial settings. Current research has successfully demonstrated correlations between flock-level optical flow patterns and key welfare indicators, particularly those related to locomotion and mortality. However, several research gaps must be addressed to leverage this technology fully. Future research should focus on exploring the potential of Optical Flow to predict a broader range of welfare indicators, including early disease detection, stress levels, and thermal comfort. It is also crucial to conduct more comprehensive studies that evaluate the effectiveness of Optical Flow across diverse commercial housing systems, management practices, and geographical locations.

This study evaluated Optical Flow and the Unrest Index independently and does not propose integrating these methods into a single hybrid model. The results highlight the differences between the two approaches. Optical Flow offers rapid, continuous flock-level movement analysis based on the difference in pixel-by-pixel brightness between two frames [23]. In contrast, the Unrest Index is based on the Hausdorff distance, which is calculated from the centroids of the hens between two frames, to evaluate the movement of the birds [29].

The Optical Flow approach does not differentiate the objects of interest in the scenes, so it is much more sensitive to variations in the brightness of the equipment and other objects in the scene. Such a characteristic was observed in our results, where the swing of the feeder and the camera’s swing interfered with the observed values. However, the equivalence of the measurements with the other two approaches added to its ease of application and low computational cost, making it a suitable tool for monitoring the movement of flocks of chickens.

The Unrest Index only requires the location of the centroids of the hens. These coordinates can be obtained through several object recognition or image segmentation methods [29,42]. In this study, we used the centroids obtained from the bounding boxes of the birds detected via the YOLO model, but it is worth noting that another method could be used. The need to detect birds in scenes makes this tool more accurate than Optical Flow but with a higher application cost. However, there is no need to identify and track the chickens; it is only necessary to recognize their location in the frames. Finally, tracking chickens using YOLO tends to be the most accurate method since, in theory, it allows for the accumulation of distances traveled by each monitored chicken [15]. We emphasize that this approach would be the most accurate if there were no loss of identification of the chickens due to occlusion of the equipment or in other situations when they are removed from the filming area [15,22]. Considering that identifications are lost, this study summarized the distances traveled for the group of chickens and, therefore, lost individual information. It also compared this method for flock monitoring with the other two evaluated methods. Notably, in the absence of an ideal method, strategies for using the available methods for assessing chicken welfare are equally important in the context of precision livestock farming [22].

These distinct tools can enhance the flexibility and robustness of welfare monitoring strategies within precision livestock farming systems, allowing producers to select the most appropriate method based on specific operational needs and technological constraints. Combining Optical Flow with other sensing technologies and AI-driven analytics offers a promising avenue for developing more holistic and robust welfare monitoring systems.

Future research could benefit from integrating thermal imaging and real-time temperature monitoring to deepen the analysis of environmental influences on broiler behavior. Thermal cameras would allow for detecting microclimate variations within the housing environment, identifying localized areas of heat accumulation or inadequate ventilation. By combining thermal data with Optical Flow and Unrest Index metrics, it would be possible to distinguish behavior changes driven by environmental stressors, such as heat stress, from those caused by internal factors like lameness. Moreover, fusing visual and thermal data through machine learning models could enhance predictive welfare assessments, enabling earlier and more targeted interventions to improve flock health and productivity.

Furthermore, more extensive longitudinal studies that track broiler welfare throughout their lifespan in commercial environments are needed. Finally, a deeper investigation into the specific behavioral correlates of different Optical Flow metrics at both the individual and flock levels is essential for improving the accuracy and interpretability of this technology. Addressing these research gaps will pave the way for developing more effective tools to improve broiler welfare and promote sustainable poultry production.

## 5. Conclusions

This study demonstrated that Optical Flow and the Unrest Index are robust, effective, and essentially equivalent methods for non-invasive monitoring of chicken welfare. Both approaches significantly correlate with walking distance (YOLO), validating their potential as automated alternatives to manual welfare assessments. Although Optical Flow exhibited minor limitations due to camera instability and environmental artifacts, it remains a promising tool for scalable and cost-effective welfare monitoring in commercial poultry systems. The Unrest Index, which relies on the centroid locations of birds between frames, was calculated using YOLO-based detection in this study. However, it can be adapted to use centroids derived from other computer vision methods, enhancing its flexibility.

The evaluated methods can be used with advanced machine learning analytics to transform poultry welfare management from reactive interventions to proactive and predictive care. Future research should focus on improving these techniques through artifact mitigation, strain-specific behavior analysis, and application in diverse housing environments. This technology-driven approach has the potential to significantly enhance animal welfare, operational efficiency, and sustainability in modern poultry production.

## Figures and Tables

**Table 1:** Pearson’s linear correlation tests of the variables resulting from the Optical Flow analysis with the distances traveled and the Unrest Index for each strain studied.

Commercial Strain	Measures	Optical Flow Variables			
		Average	Variance	Skewness	Kurtosis
Cobb^®^	Distance	0.973	0.941	−0.909	−0.916
	Unrest Index	0.924	0.944	−0.841	−0.862
Ross^®^	Distance	0.913	0.815	−0.859	−0.519
	Unrest Index	0.956	0.939	−0.775	−0.419
Hybro^®^	Distance	0.910	0.686	−0.815	−0.226
	Unrest Index	0.856	0.681	−0.730	−0.247

**Table 2:** Advantages, disadvantages, and recommended use context of each method of assessing the flock’s movement.

Method	Advantages	Disadvantages	Recommended Use Context
Walking distance (YOLO)	Deterministic mathematical method. Accurately detects chickens, even in crowded conditions. Fast processing once the model is trained.	Depends on maintaining bird identification to accumulate per-run distance. High human and computational costs for model training.	YOLO-based walking distance is recommended for high-precision flock studies where individual tracking is critical. One must be aware of the challenge of maintaining identification for correct tracking.
Unrest Index	Deterministic mathematical method. Can use any method to detect chicken centroids. Does not require model training as long as the centroids are available.	Must be carried out in two stages. Depending on the quality of the segmentation and the agglomeration of the birds, it may be imprecise.	The Unrest Index is suggested for scenarios where centroid detection is feasible but individual tracking is not mandatory.
Optical Flow	Can be performed in one step. No model training required. Provides four metrics that can more clearly indicate the variability of movement among monitored individuals.	Captures the movement of the equipment and other objects in the scene. Captures false movement caused by camera instability.	Optical Flow is recommended for quick deployment in commercial settings where equipment stability is not challenging and group-level monitoring suffices.

## Data Availability

Data will be available upon request to the corresponding author.

## Supplementary Materials

The following supporting information can be downloaded at https://www.mdpi.com/article/10.3390/ani15091311/s1, Raw data S1: Raw data containing the values of Unrest Index and Optical Flow metrics obtained frame by frame during the processing of the videos used in this study.
